# Safety and efficacy of probiotic supplements as adjunctive therapies in patients with COVID-19: A systematic review and meta-analysis

**DOI:** 10.1371/journal.pone.0278356

**Published:** 2023-03-31

**Authors:** Julie Zhu, Tyler Pitre, Carmen Ching, Dena Zeraatkar, Steven Gruchy

**Affiliations:** 1 Department of Medicine, Division of Digestive Care and Endoscopy, Dalhousie University, Halifax, Nova Scotia, Canada; 2 Department of Medicine, McMaster University, Hamilton, Ontario, Canada; 3 Faculty of Medicine, Dalhousie University, Halifax, Nova Scotia, Canada; 4 Department of Anesthesia, McMaster University, Hamilton, Ontario, Canada; 5 Department of Health Research Methods Evidence and Impact, McMaster University, Hamilton, Ontario, Canada; University of Catanzaro: Universita degli Studi Magna Graecia di Catanzaro, ITALY

## Abstract

**Background and aims:**

Oral probiotic supplementation may be a beneficial adjunctive therapy for patients with symptomatic COVID-19. However, its safety and efficacy are unclear. We aimed to investigate how probiotic supplementation impacts COVID-19 symptom trajectory and patient outcomes by conducting a systematic review and meta-analysis of randomized controlled trials (RCTs).

**Methods:**

RCTs randomizing patients with COVID-19 to probiotics were searched in PubMed Central, Embase, CINAHL, and Cochrane Library from inception to July 31, 2022. We performed a random-effects pairwise meta-analysis for all outcomes using the restricted maximum likelihood (REML) estimator. We used the GRADE approach to assess the certainty of the evidence.

**Results:**

A total of 1027 participants from eight RCT studies were included in the meta-analysis. Probiotic supplements probably reduce the incidence of diarrhea (RR 0.61 [0.43 to 0.87]; moderate certainty) and probably reduce cough or dyspnea compared to placebo/standard care (RR 0.37 [0.19 to 0.73]; moderate certainty). Probiotic supplements may improve composite endpoint measured by clinical escalation or mortality compared to placebo (RR 0.41 [0.18 to 0.93]; low certainty evidence); however, they may not significantly reduce the need for clinical escalation (RR 0.57 [0.31 to 1.07]; low certainty evidence) or mortality (RR 0.50 [0.20 to 1.29]; low certainty evidence). In addition, the probiotic supplement is associated with reduced adverse events (RR 0.62 [0.46 to 0.83]; moderate certainty).

**Conclusion:**

Early probiotic supplement is a safe and effective adjunctive therapy that reduces the risk of symptoms and health care burden related to COVID-19 across all severity types.

## Introduction

Over 628,694,943 COVID-19 cases and over 6,576,088 associated deaths have been confirmed since the coronavirus disease 2019 (COVID-19) pandemic began [1]. Despite 12,961,382,558 vaccine doses administered globally and COVID-19-specific treatments, disease burden, morbidity, and mortality remain significant, resulting in ongoing pandemic waves in many regions [1]. Symptomatic illness rate remains high at 37,764 per 100,000 across all ages, and hospitalization rate and mortality have been rising from time to time, disproportionally impacting those with weaker immune systems and older populations above 65 [2]. Respiratory complaints and complications from gastrointestinal symptoms are among the frequent causes of emergency room visits and hospitalization in patients with COVID-19 [3,4]. GI symptoms such as diarrhea, abdominal pain, loss of appetite, nausea or vomiting can emerge in parallel or earlier than a dry cough, fever or dyspnea [4]. More than 50% of hospitalized patients reported at least one gastrointestinal (GI) symptom. Up to 20% of all COVID-19 cases were manifested by GI symptoms alone and may be associated with longer disease duration and/or severity [4,5]. This has prompted investigations for well-tolerated and cost-effective therapies that effectively reduce disease severity and symptom burden among patients and potentially decrease healthcare resource utilization. Probiotic therapy or bacteriotherapy is an oral supplement of live microorganisms with multiple health benefits. It exerts beneficial properties by enhancing intestinal microbial homeostasis and modulating the host’s immune response to pathogens [6]. Altered gut homeostasis, or dysbiosis, is associated with various intestinal and extra-intestinal chronic diseases such as rheumatoid arthritis, osteoporosis and diabetes [7–10]. Furthermore, the immunomodulating roles of gut microflora in lung homeostasis and lung disease underscore the gut-lung axis crosslinked by pathogen-associated molecular patterns (PAMPs), lipopolypsaccharide (LPS) and migration of immune cells from the gut to the lungs [11]. Probiotic use is associated with fewer episodes of acute upper respiratory tract infections with no significant side effects [12]. Several strains of probiotics studied in randomized controlled trials effectively prevented ventilator-associated bacterial pneumonia in critically ill patients on mechanical ventilation [13,14]. Recently published systematic review and meta-analysis in critically ill patients demonstrated a significant role of probiotics in reducing ventilator and healthcare-associated pneumonia to decrease intensive care unit (ICU), hospital length of stay and utilization of invasive mechanical ventilation based on low-certainty evidence [15]. From the gastrointestinal aspect, several studies showed probiotics prevented and decreased the onset of antibiotic-associated diarrhea (AAD) [16] and antibiotic-associated C. difficile colitis, potentially reducing healthcare resource utilization [17–19].

The antiviral properties of probiotics were demonstrated by in vitro studies for their ability to inhibit viral infection and replication, and suppress proinflammatory cascade [20,21]. SARS-Cov-2 infection of human epithelial cells was attenuated by Lacticaseibacilus, and pro-inflammatory gene expression was similarly suppressed. In another in vitro study, Lactiplantibacillus effectively suppressed the replications of SARS-Cov-2 in human respiratory epithelial cells [20,21]. The clinical application probiotics was studied in a propensity-score matched retrospective study, Bifidobacterium, Lactobacillus and Enterococcus associated with shorter recovery time and reduced hospitalization days in COVID-19 patients with moderate to severe symptoms [22]. More compelling evidence came from several randomized controlled trials involving probiotic supplements during the coronavirus disease 2019 (COVID-19) pandemic. However, there is currently a lack of higher-level evidence evaluating probiotic therapy’s potential impact on COVID-19 symptoms and disease course.

We aimed to conduct a systematic review and meta-analysis of randomized controlled trials to investigate the safety and efficacy of probiotic therapy in patients diagnosed with COVID-19 on their symptom development and overall clinical outcomes.

## Methods

We pre-registered a protocol on PROSPERO (International prospective register of systematic reviews): CRD42022328256. We report our results according to the PRISMA checklist [23].

### Eligibility criteria

We included all randomized controlled trials (RCTs) that randomized hospitalized or outpatient individuals with symptomatic COVID-19 infection with a confirmed diagnosis via a positive COVID-19 test (i.e., Real Time-Polymerase chain reaction or point of care testing). In addition, we included RCTs that randomized patients to oral probiotic supplementation immediately before or after the study period, compared to placebo or standard care. The probiotic agents included are prescribed at a standard, recommended, therapeutic dosage or adaptive dosing with appropriate clinical reasoning. The probiotic agent does not need to be licensed by the Food and Drug Administration (FDA) to be included. On top of the probiotic agent, other adjuncts, such as nutritional supplements or medication, can be present if they are in all arms of the study. Primary outcomes included reported adverse events related to treatments, any COVID-19 symptom, its duration, and severity with a focus on gastrointestinal and respiratory symptoms and change in COVID-19-related biomarkers. Secondary outcomes included escalation of care (requiring oxygen support by nasal cannula, non-invasive mask, invasive mechanical ventilation, circulatory support, or vasopressor use) and/or deaths. We excluded studies examining non-COVID-19 coronavirus-related respiratory illnesses such as SARS-CoV/ MERS-CoV and studies whereby the COVID-19 population is strictly pediatric (< 18 years old).

With the assistance of an experienced librarian, we searched PubMed Central, Embase, CINAHL, and Cochrane Library from inception to July 31, 2022, for randomized trials meeting our inclusion criteria. Search string details are provided in (S1 Table). In addition, trial registers were searched at ClinicalTrials.gov, the Cochrane Central Register of Controlled Trials and PROSPERO for ongoing trial reports. S1 Table presents our database search strategy. There was no year or language restriction.

### Study selection

Following training and calibration exercises to ensure sufficient agreement, pairs of reviewers, working independently and in duplicate, screened titles, and abstracts of search records and, subsequently, the full texts of records deemed potentially eligible at the title and abstract screening stage. Reviewers resolved discrepancies by discussion and, when necessary, by adjudication with a third party.

### Data collection

Following training and calibration exercises to ensure sufficient agreement, pairs of reviewers, working independently and in duplicate, collected data on general information (first author, publication year, the country in which the study was conducted), trial characteristics in PICO format (participant setting; details of study design, probiotic microorganisms, and their frequency, route of administration and dosage, as well as the duration of treatment; control via placebo and/or standard or care treatment; primary and secondary outcomes, the total length of follow-up), patient characteristics (average age, sex, COVID-19 severity classification according to WHO COVID-19 Clinical Progression Scale [24]), and outcomes of interest (as previously described). In trials with information on clinical escalation and deaths, we defined a single composite outcome by tallying the number of both endpoint events while subtracting the number of reported deaths from the clinical escalation subgroups to avoid concomitant events or double counting. In addition, we sought clarifications from the corresponding authors regarding patient characteristics, protocols, and other unpublished data whenever necessary.

### Risk of bias

Following training and calibration exercises to ensure sufficient agreement, pairs of reviewers, working independently and in duplicate, assessed the risk of bias with the Cochrane tool for assessing the risk of bias in randomized trials (RoB 2.0). We assessed the risk of bias across five domains: bias arising from the randomization process, bias due to departures from the intended intervention; bias from missing outcome data; bias in the measurement of the outcome; and bias in the selection of the reported result. Reviewers resolved discrepancies by discussion and, when not necessary, with adjudication by a third-party reviewer.

### Data analysis

We performed a random-effects pairwise meta-analysis for all outcomes, using the inverse variance method with a restricted maximum likelihood estimator (REML). When convergence was not possible, we used the maximal likelihood estimator. We also planned to perform a fixed-effect analysis for estimates with less than four trials. We reported dichotomous outcomes using relative risk (RR) with a 95% confidence interval (CI) and continuous outcomes as mean differences with 95% CI.

To optimize interpretability, for dichotomous outcomes, we calculated absolute effects by multiplying relative effects with the median baseline risk calculated from the placebo arm of the randomized trials. As a result, we presented an absolute risk difference and associated 95% CI.

We performed subgroup analysis based on patient setting (outpatient versus inpatient). We hypothesized there may be differences in the effect of probiotics on clinical symptoms based on whether probiotics were administered in the outpatient versus inpatient setting. For statistically significant subgroups, we used the Instrument for assessing the Credibility of Effect Modification Analyses (ICEMAN) tool to assess the credibility of the subgroups [25].

For outcomes with ten or more trials, we assessed for publication bias by visual inspection of funnel plots and Egger’s test.

All data were analyzed using STATA version 17.0

### Certainty of the evidence

Two reviewers, working independently and in duplicate, assessed the certainty of the evidence using the GRADE approach.

We made judgments of imprecision using the minimally contextualized approach. A minimally contextualized approach considers whether confidence intervals include the minimally important difference and thus does not consider whether it includes both minimally important and large effects. We sourced minimally important differences (MID) from available literature and by consensus when formal minimally important differences were not available in the literature. Whether a probiotic treatment is effective depends on whether the point estimate includes the MID.

We report our results using the standard language guidance for GRADE. This involves referring to the evidence’s certainty by using specific modifiers. For example, the effectiveness of treatment could be rated by saying: Treatment X reduces mortality (high certainty), Treatment X probably reduces mortality (moderate certainty), Treatment X may reduce mortality (low certainty), and the Effect of Treatment X on mortality is very uncertain. We rated the certainty for each comparison and outcome as high, moderate, low, or very low based on the risk of bias, inconsistency, indirectness, publication bias, and imprecision.

## Results

### Study selection

**Fig 1:**
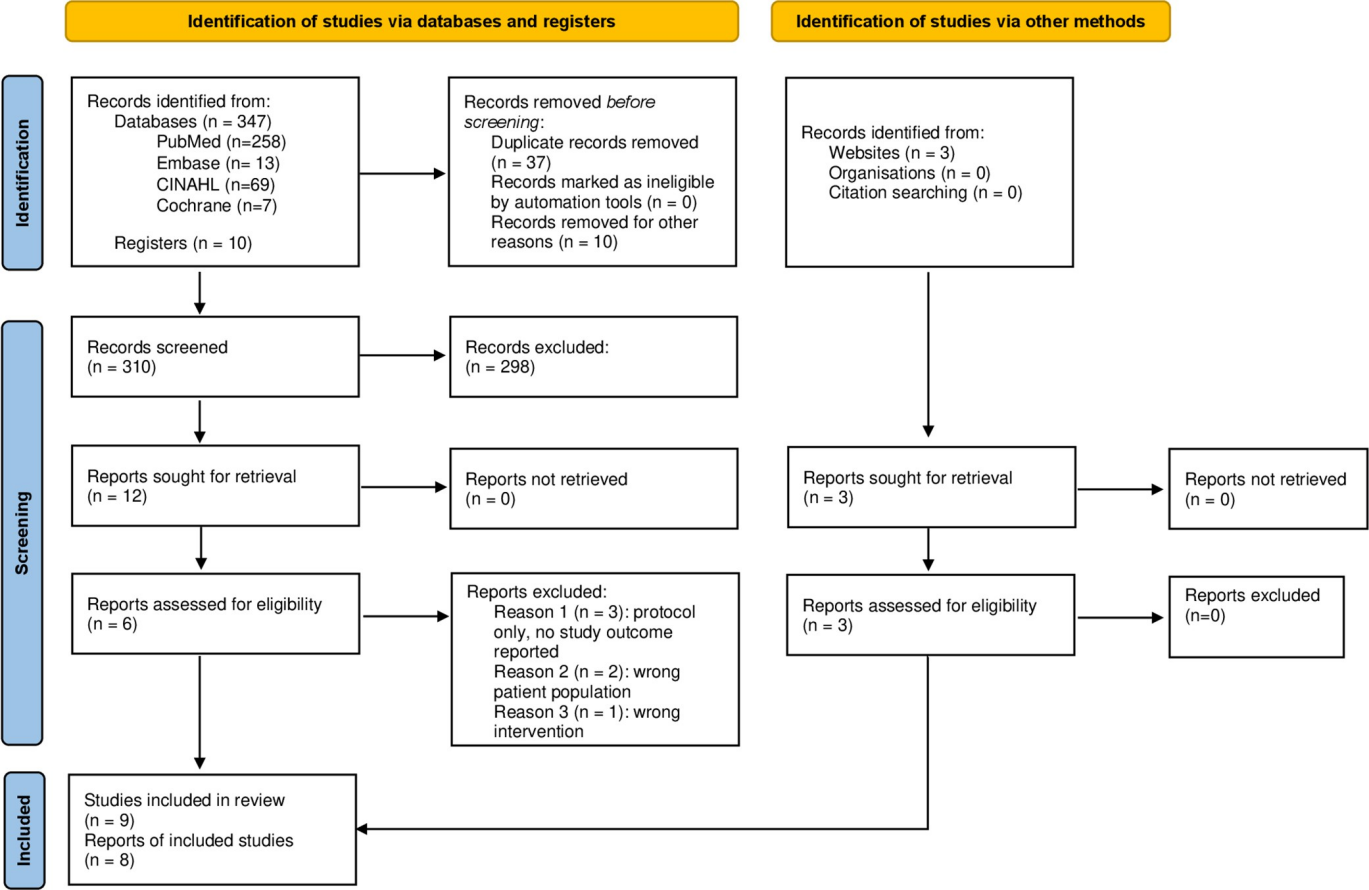
PRISMA flow diagram.

We identified 323 unique studies to screen for eligibility, and 15 were eligible for full-text review. Nine studies met the inclusion criteria for a systematic review and underwent data extraction [26–34]; eight were eligible to be included in the final meta-analysis [26–33]. Fig 1 presents more detail on the study selection process.

### Study characteristics

We included nine studies that randomized COVID-19 patients to probiotics and eight reported outcomes of interest. In the eight studies that were included in the meta-analysis, there were a total of 1027 participants. The mean age was 57.2 years, and 49.2% were male. Probiotics were administered by an oral route. Frequency, dosage, and duration of probiotic administration are summarized in the supplementary (S3 Table). Five studies involved multi-strain bacterial formulations, two involved a single bacterial strain and one involved a single-strain yeast formulation. They were all conducted on patients with confirmed COVID-19 diagnoses. In addition, two studies were conducted in outpatient settings in patients with mild symptoms.

In comparison, six studies took place in hospitals and recruited patients with moderate to severe symptoms according to the WHO classification [24]. In all studies, probiotics were given immediately or within 48 hours of COVID-19 diagnosis. The duration of probiotic treatment was between 6 to 30 days, with once or split daily dosing regimens.

S2 and S3 Table present more details on the characteristics of the included studies (systematic review N = 9; meta-analysis N = 8).

### Risk of bias in studies

**Fig 2:**
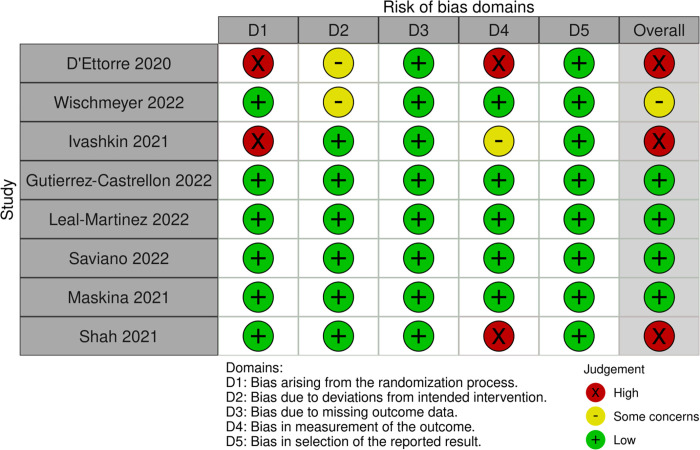
Risk of bias assessment.

Two trials were at risk of bias due to concerns with allocation concealment, and two were at risk of bias due to concerns around the measurement of the outcome; no trial was at risk of bias due to missing data or selective reporting of results. Fig 2 presents more details on the risk of bias in included trials.

### Outcomes

#### Cough or dyspnea-like respiratory symptoms

Five trials reported respiratory symptoms of cough or dyspnea-like reparatory symptoms, including 658 patients with a median follow of 14 days. Probiotics probably improve cough or dyspnea compared to placebo/standard care (RR 0.37 [0.19 to 0.73]; moderate certainty). We rated down once for imprecision. There was unimportant heterogeneity (I^2^ = 38.11%).

**Fig 3:**
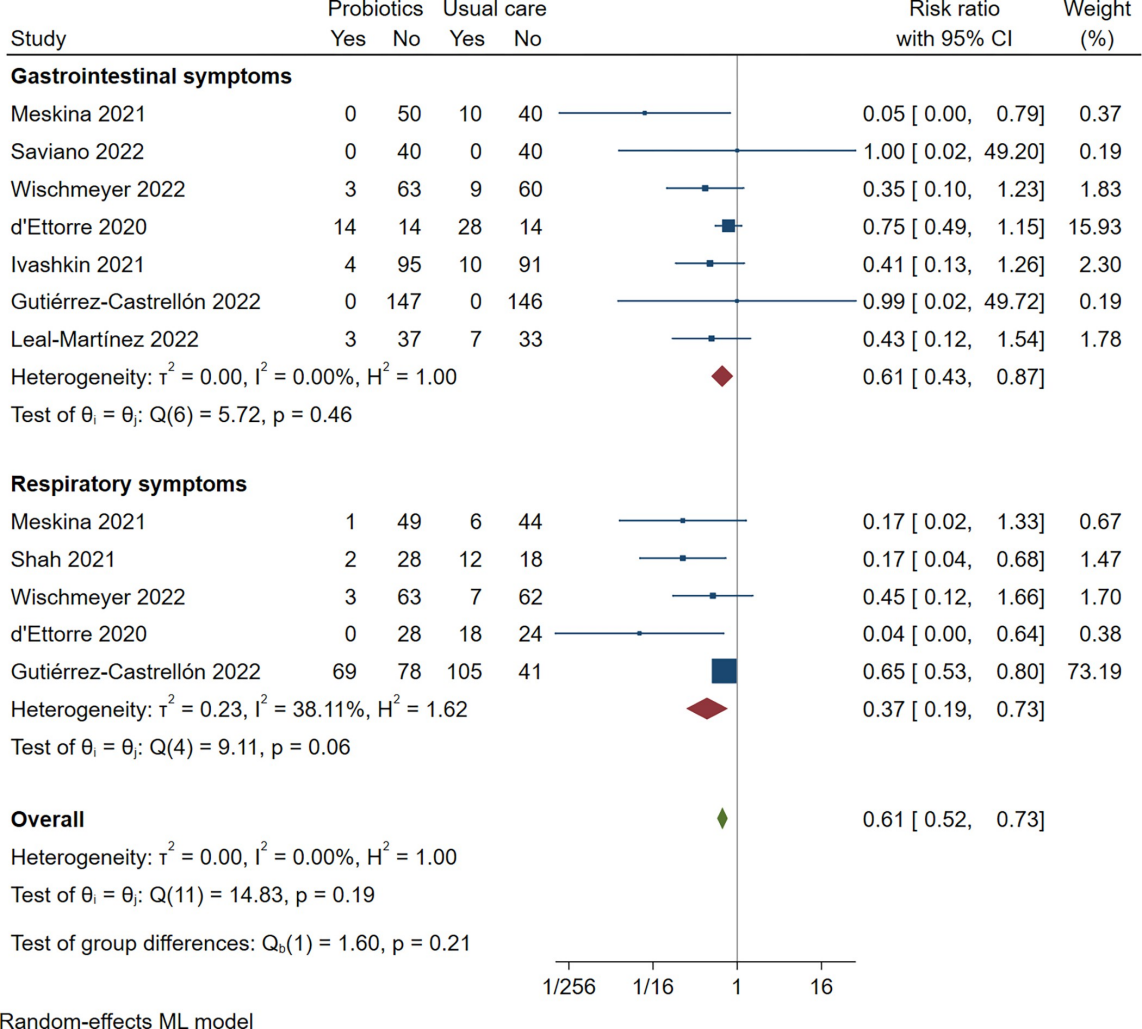
Gastrointestinal and respiratory symptoms.

Table 1 reports the summary of the findings. Fig 3 presents the forest plot.

**Table 1:** Rating the quality of evidence by the GRADE approach.

Outcomes	№ of participants (studies) Follow-up	Certainty of the evidence (GRADE)	Relative effect (95% CI)	Anticipated absolute effects		
				Risk with Usual care	Risk difference with Probiotics	
**Mortality**	823 (6 RCTs)	⨁⨁◯◯ Low^a^^,^^b^	**RR 0.50** (0.20 to 1.29)	38 per 1,000		**19 fewer per 1,000** (31 fewer to 11 more)
**Composite clinical escalation and death**	823 (6 RCTs)	⨁⨁◯◯ Low	**RR 0.43** (0.19 to 0.94)	246 per 1,000		**140 fewer per 1,000** (199 fewer to 15 fewer)
**Clinical escalation**	823 (6 RCTs)	⨁◯◯◯ Very low^a^^,^^b^	**RR 0.57** (0.31 to 1.07)	208 per 1,000		**89 fewer per 1,000** (143 fewer to 15 more)
**Gastrointestinal symptoms**	958 (7 RCTs)	⨁⨁⨁◯ Moderate	**RR 0.61** (0.43 to 0.87)	131 per 1,000		**51 fewer per 1,000** (75 fewer to 17 fewer)
**Respiratory symptoms**	658 (5 RCTs)	⨁⨁⨁◯ Moderate	**RR 0.37** (0.19 to 0.73)	439 per 1,000		**277 fewer per 1,000** (356 fewer to 119 fewer)
**Adverse events**	938 (7 RCTs)	⨁⨁⨁◯ Moderate	**RR 0.62** (0.46 to 0.83)	165 per 1,000		**63 fewer per 1,000** (89 fewer to 28 fewer)
**Change in CRP**	340 (3 RCTs)	⨁◯◯◯ Very low^b^	-	The mean change in CRP was **0** mg/L		MD **1.22 mg/L lower** (3.52 lower to 1.09 higher)

GRADE working group grades of evidence.

High certainty: We are very confident that the true effect lies close to that of the estimate of the effect.

Moderate certainty: We are moderately confident that the effect estimate (the true estimate is likely to be close to the estimate of the effect, but there is a possibility that it is substantially different).

Low certainty: Our confidence in the effect estimate is limited (the true effect may be substantially different from the estimate of the effect).

Very low certainty: We have very little confidence in the effect estimate (the true effect is likely to be substantially different from the estimate of effect).

Cl, confidence interval; MD, mean difference; RR, risk ratio.

*the risk in the intervention group (and its 95% Cl) is based on the assumed risk in the comparison group and the relative effect of the intervention (and its 95% CI).

^a^composite outcome.

^b^includes MID.

### Gastrointestinal symptoms

Seven trials reported gastrointestinal symptoms of diarrhea, including 958 patients with a median follow of 26 days. Probiotics probably improve the risk of diarrhea in COVID-19 patients (RR 0.61 [0.43 to 0.87]; moderate certainty). We rated the down once for imprecision. There was no heterogeneity (I^2^ = 0.00%)

### Table 1 reports the summary of the findings. Fig 3 presents the forest plot

#### Change in inflammatory biomarkers

Four trials reported c-reactive protein levels, including 630 patients with a median follow-up of 14 days. The effect of probiotics on c-reactive protein levels compared to placebo/standard care is very uncertain (mean difference -9.27 mg/L [-28.10 mg/L to 9.56 mg/L]; very low certainty). We rated down twice for imprecision and once for inconsistency. There was important heterogeneity (I^2^ = 99.89%).

Table 1 reports the summary of the findings. S7 Fig presents the forest plot.

#### Adverse events

Seven trials reported treatment-associated adverse events, including 938 patients with a median follow-up of 24 days. Probiotics probably have reduced adverse events compared to placebo/standard care (RR 0.62 [0.46 to 0.83]; moderate certainty). We rated down once for imprecision. There was no heterogeneity (I^2^ = 0.00%).

Table 1 reports the summary of the findings. S8 Fig presents the forest plot.

#### Subgroup analysis

We found a statistically significant subgroup difference for respiratory symptoms with a patient setting as a modifier (p<0.001). There was a substantially larger effect in hospitalized patients (RR 0.13 [95% CI 0.05 to 0.39]) as compared to outpatients (RR 0.65 [95% CI 0.53 to 0.79]). We found this subgroup to have moderate to low credibility using the ICEMAN tool. S9 Fig presents the forest plots.

We did not find statistically significant subgroups for mortality, clinical escalation, composite of the two, gastrointestinal symptoms, serious adverse events or change in CRP (mg/L). S1–S4 Figs present the forest plots for these analyses.

### Clinical escalation

Six trials reported clinical escalation due to COVID-19, including 823 patients with a median follow of 25 days. In the probiotic arms, 12.7% of patients underwent escalation of care versus 20.5% in the placebo arms. Probiotics may not reduce clinical escalation compared to placebo (RR 0.57 [0.31 to 1.07]; low certainty). We rated down twice for imprecision and once for risk of bias. There was unimportant heterogeneity (I^2^ = 13.4%)

**Fig 4:**
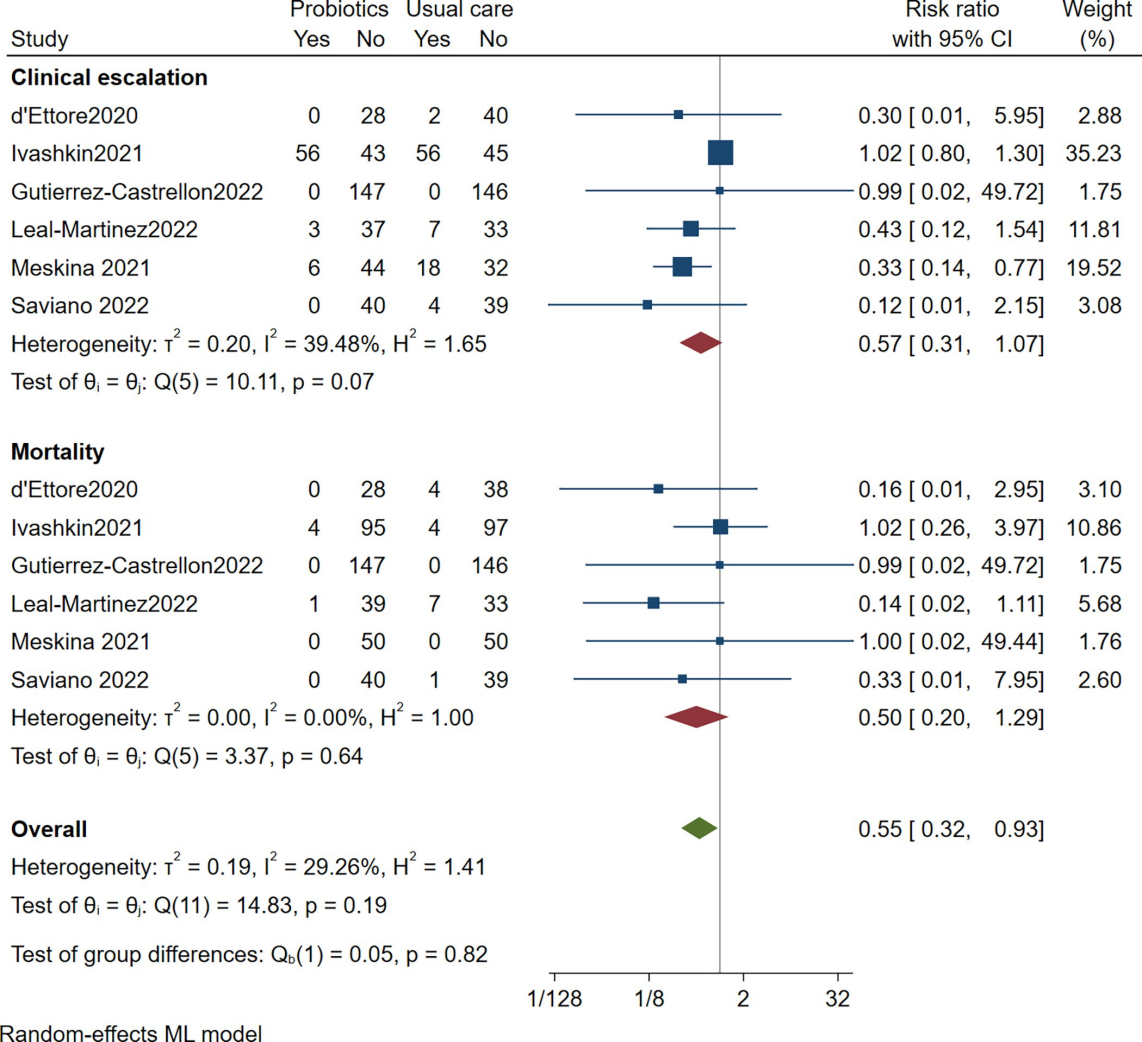
Clinical escalation and mortality.

Table 1 reports the summary of the findings. Fig 4 presents the forest plot.

### Mortality

Six trials reported deaths from COVID-19, including 823 patients with a median follow of 25 days. The mean mortality rates were 1.1% in the probiotic arm and 5.6% in the placebo arm. Probiotics may have no significant effect on mortality compared to placebo (RR 0.50 [0.20 to 1.29]; low certainty). We rated down twice for imprecision and once for risk of bias. There was no heterogeneity (I^2^ = 0.00%)

Table 1 reports the summary of the findings. Fig 4 presents the forest plot.

### Composite clinical escalation and mortality

Six trials reported clinical escalation and deaths from COVID-19, including 823 patients with a median follow of 25 days. Probiotics may reduce composite clinical escalation or mortality compared to placebo (RR 0.41 [0.18 to 0.93]; low certainty). We rated down twice for imprecision. There was significant heterogeneity (I^2^ = 64.58%).

**Fig 5:**
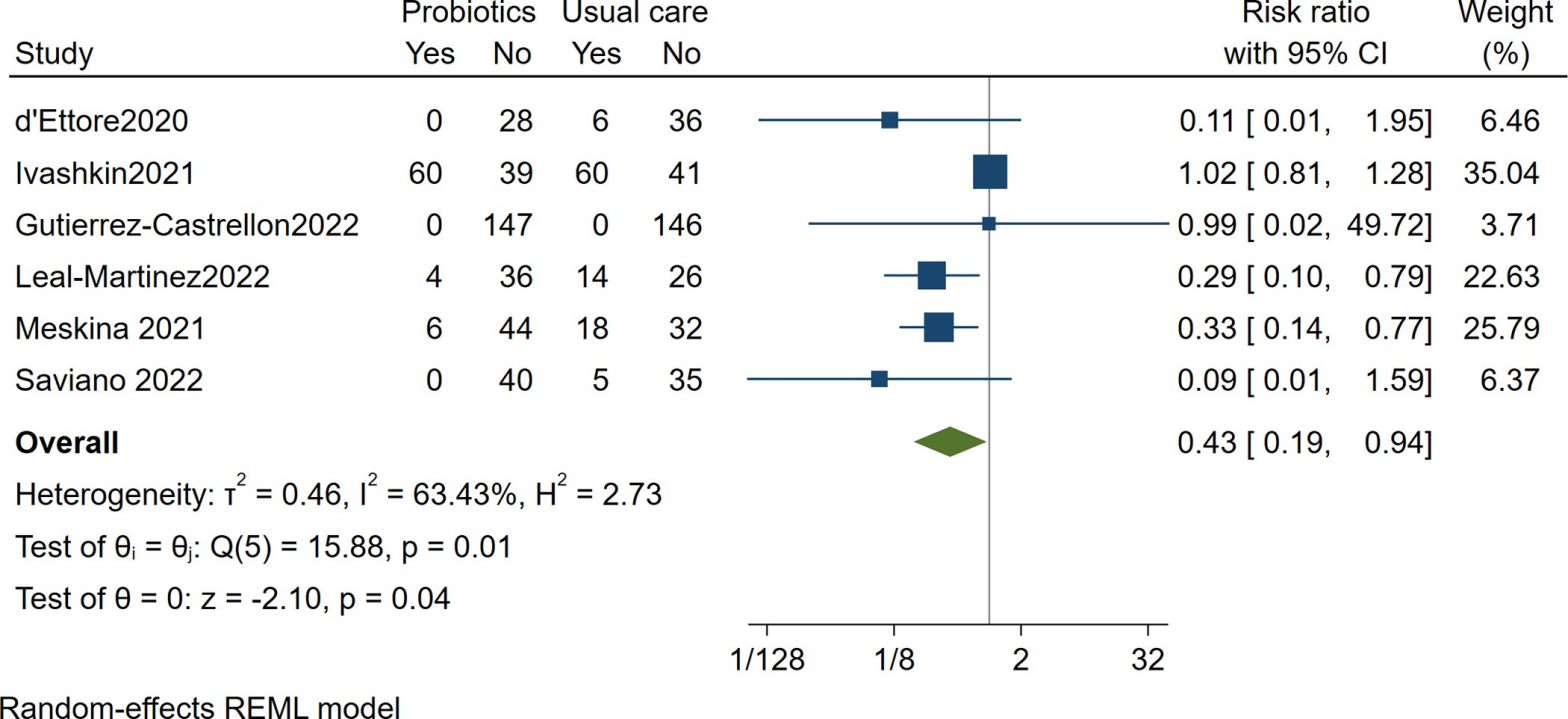
Composite clinical escalation or mortality.

Table 1 reports the summary of the findings. Fig 5 presents the forest plot.

## Discussion

### Main findings

We found that probiotics were effective in treating COVID-19 symptoms, particularly respiratory and gastrointestinal symptoms. Early probiotic supplements and standard therapy decrease gastrointestinal and respiratory symptoms and are associated with a lower risk for adverse events, confirming their safety profiles with moderate certainty of evidence. Plausible explanations for these findings are decreased COVID-19 symptom duration and severity, and reduced hospital-acquired infections such as ventilator-associated pneumonia and antibiotic-associated diarrhea in the probiotic-treated groups. The potential implications are probiotics may lead to reduced emergency room visits and hospitalization rates, reduced length of hospital or ICU stays, and other healthcare resource utilization rates among patients with COVID-19. Furthermore, reduced healthcare resource utilization is supported by lower composite end-point care escalation or death from COVID-19.

### Relation to previous findings

Currently, several COVID-19-specific treatments exist and are prioritized for selected patients with selected patient demographics and clinical profiles [35]. Potential barriers and contraindications to these therapies are relatively common and can negatively affect patient access, especially in the more vulnerable sub-populations with known adverse outcomes [36]. In outpatient settings, access to COVID-19 therapies, delayed contact with health professionals, and drug-drug interactions are significant obstacles to administering COVID-19-specific therapies [37]. At the same time, the majority of these therapies require parenteral administration. Oral probiotics, being an oral formulation, are safe, convenient and accessible options for patients of all severity types. In the inpatient settings, there were no reported adverse events related to the concomitant use of probiotics and other COVID-19 treatments suggesting probiotics were well tolerated by various inpatient regimens and caused no strain-specific nosocomial infections across the disease spectrum.

Before this review, there has yet to be a systematic review and meta-analysis generated from randomized controlled trials on the effects of probiotics on COVID-19 symptoms and disease course. Previous reviews were qualitative and retrospective [38,39]. However, there were recent systematic reviews and meta-analyses on probiotics in hospital-acquired and ventilator-associated pneumonia [40,41]. These reviews have provided evidence for reduced ventilator-associated pneumonia (VAP) in non- COVID-19 patients treated with probiotics. Patients with COVID-19 had an increased risk of VAP and hospital-acquired pneumonia compared to non-COVID patients admitted to ICU or hospitals and overall experienced considerable morbidity and mortality [42]. Speculated intrinsic factors are more severe parenchymal lung damage and poorer lung compliance from COVID-19 than non-COVID patients, and extrinsic factors included increased use of systematic immunosuppressants. This systematic review provides the best-updated evidence for the effects of probiotics on symptomatic COVID-19 cases, among them those with respiratory symptoms. Patient settings modified the respiratory effect. Hospitalized patients with moderate to severe respiratory symptoms experienced greater risk reduction than those treated in outpatient settings. In addition to antiviral properties, the results suggested probiotics may effectively reduce ventilator-associated pneumonia and hospital-acquired pneumonia among COVID-19 populations.

Lower gastrointestinal symptoms such as diarrhea may be present in 34% of COVID-19 cases, and abdominal pain or nausea in 26% to 35% [43]. SARS-CoV-2 RNA can be readily detected in stool. Although the mechanisms are still poorly understood, disruption of the intestinal microbial composition by SaRS-Cov-2 and antibiotics used to treat COVID-19 may worsen dysbiosis and inflammation of the respiratory and gastrointestinal systems [44,45]. The results from this review are consistent with previous systematic reviews and meta-analyses, whereby probiotic therapies were shown to prevent antibiotic-associated diarrhea and reduce acute viral-associated diarrhea symptoms [45].

The lack of effect on mortality is consistent with reports from retrospective and non-randomized prospective studies [46] and results from non-COVID critically ill patients [40].

This review focuses on probiotic safety as an adjunctive therapy to existing therapies such as dexamethasone, antibiotics, anticoagulants, and other types of COVID-19-specific treatments, including remdesivir and immunomodulators. There were significantly fewer adverse event rates from patients on probiotics; in particular, there were no reports of bacterial infections from probiotic therapy, regardless of comorbidity and disease severity.

### Implications and future directions

One of the most robust findings was the reduction in COVID-19 symptoms in the probiotic groups. Probiotics may be recommended as a cost-effective strategy for patients with COVID-19 symptoms to prevent symptom progression and decrease symptom duration. The mechanism of action for probiotics and their effectiveness in long COVID symptoms warrant investigation by future studies; there may be differences in response rate and response magnitude in specific subpopulations that necessitate future investigations.

### Strengths and limitations

Our review has several strengths. First, our protocol was prospectively registered on PROSPERO. Second, the meta-analysis is generated from randomized controlled trials involving patients with a confirmed diagnosis of COVID-19 by nasopharyngeal PCR, with a low risk of bias for the majority; this strengthens its internal validity. Third, we have included populations from outpatient and inpatient settings with a spectrum of disease severity. Fourth, the populations examined by our study are diverse in countries of origin, age, and ethnicity, thus lending support to its external validity. Finally, we performed sub-group analysis based on patient settings to explore how sub-populations with different clinical characteristics may respond to probiotics.

Our review has a few limitations. First, probiotic supplementations are somewhat diverse, and their mechanism of action needs to be better understood; externalizing our findings to other types of probiotics may be too ambitious. Second, there is limited data on older populations more vulnerable to symptomatic COVID-19 infections and sequelae; sub-group analysis based on patient demographics, such as age, may clarify who will benefit the most from probiotic therapy. Third, primary or secondary preventative roles are beyond this review’s scope since only confirmed cases are included. Lastly, given that some trials were published during the early-to-mid pandemic era, there could be selection and publication bias.

## Conclusion

Our study found favorable effects from the early probiotic supplements in patients treated for COVID-19 in hospital or outpatient settings. An early probiotic supplement is safe in mild, moderate, or severe disease types and is associated with reduced symptom progression and duration. In addition, the evidence suggests that probiotic supplements may play a role in reducing overall health costs associated with COVID-19 by decreasing its disease burden.

***Registration*:** Protocol was registered on PROSPERO (International prospective register of systematic reviews): CRD42022328256.

## Supporting information

S1 Checklist PRISMA 2020 checklist. (DOCX)

S1 Fig Subgroup analysis of clinical escalation based on patient setting. (TIFF)

S2 Fig Subgroup analysis of composite end point based on patient setting. (TIFF)

S3 Fig Subgroup analysis of CRP level (mg/L) based on patient setting. (TIFF)

S4 Fig Subgroup analysis of gastrointestinal symptoms based on patient setting. (TIFF)

S5 Fig Subgroup analysis of mortality based on patient setting. (TIFF)

S6 Fig Subgroup analysis of adverse events based on patient setting. (TIFF)

S7 Fig C-reactive protein levels (mg/L). (TIFF)

S8 Fig Adverse events. (TIFF)

S9 Fig Subgroup analysis of respiratory symptoms based on patient setting. (TIFF)

S1 Table Database search strategies. (TIFF)

S2 Table Outcome summary. (TIFF)

S3 Table Study characteristics. (TIFF)
