# Supplementary figures and images for: Safety and efficacy of probiotic supplements as adjunctive therapies in patients with COVID-19: A systematic review and meta-analysis
Source: PLoS One. 2023 Mar 31;18(3):e0278356. doi: 10.1371/journal.pone.0278356 (PMC10065254; doi:10.1371/journal.pone.0278356)

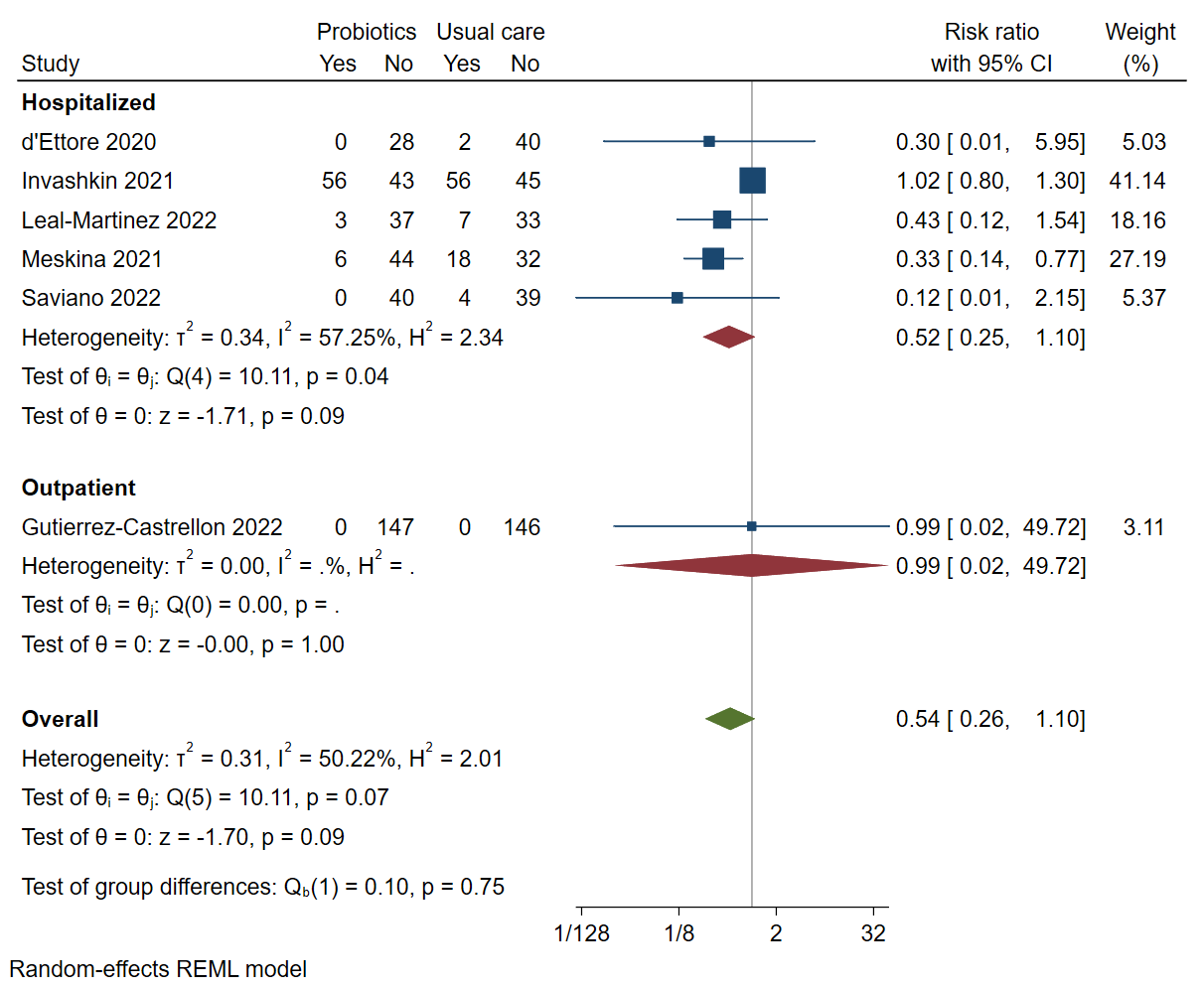

Supplement: S1 Fig — (TIFF) [file pone.0278356.s002.tiff]

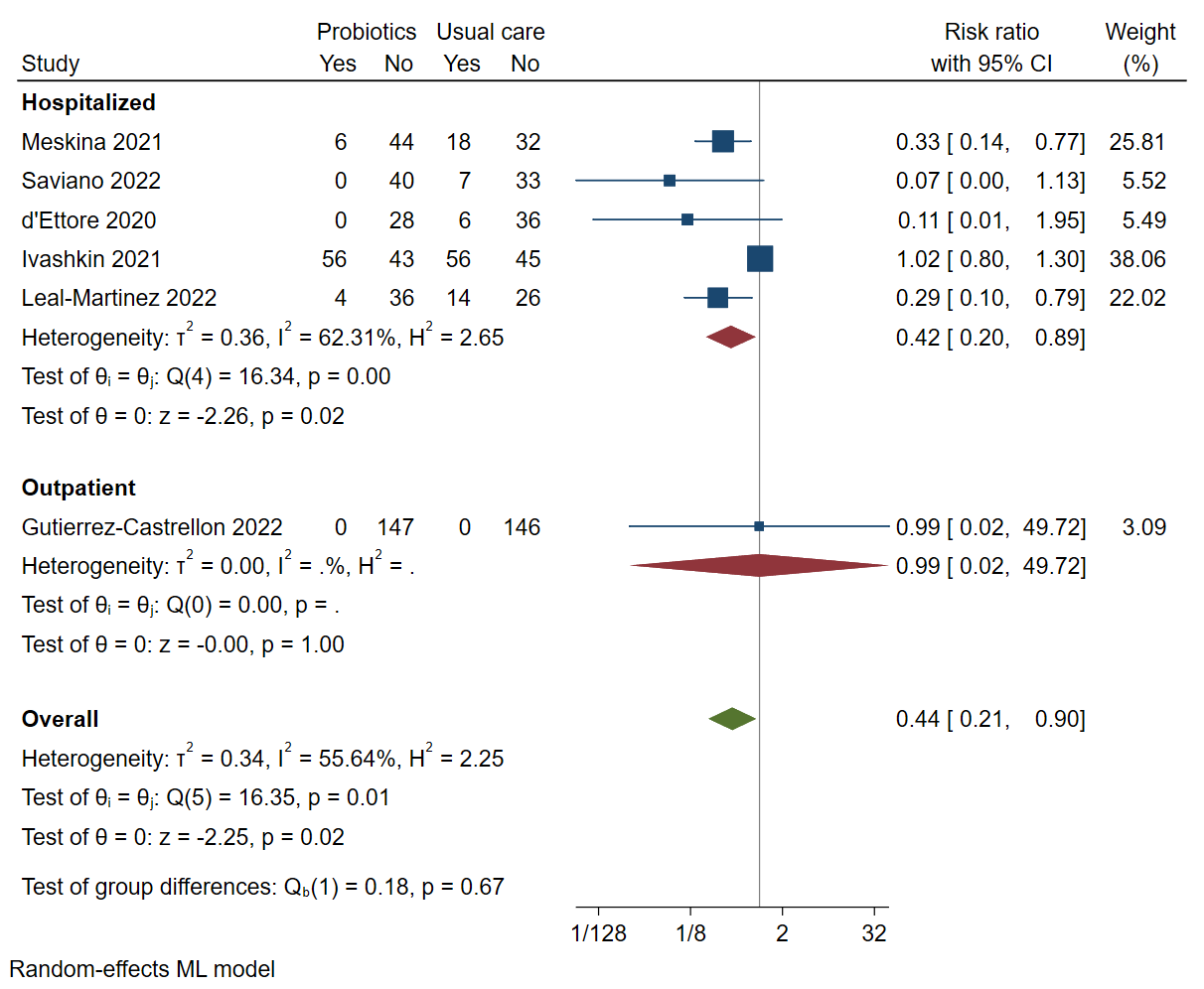

Supplement: S2 Fig — (TIFF) [file pone.0278356.s003.tiff]

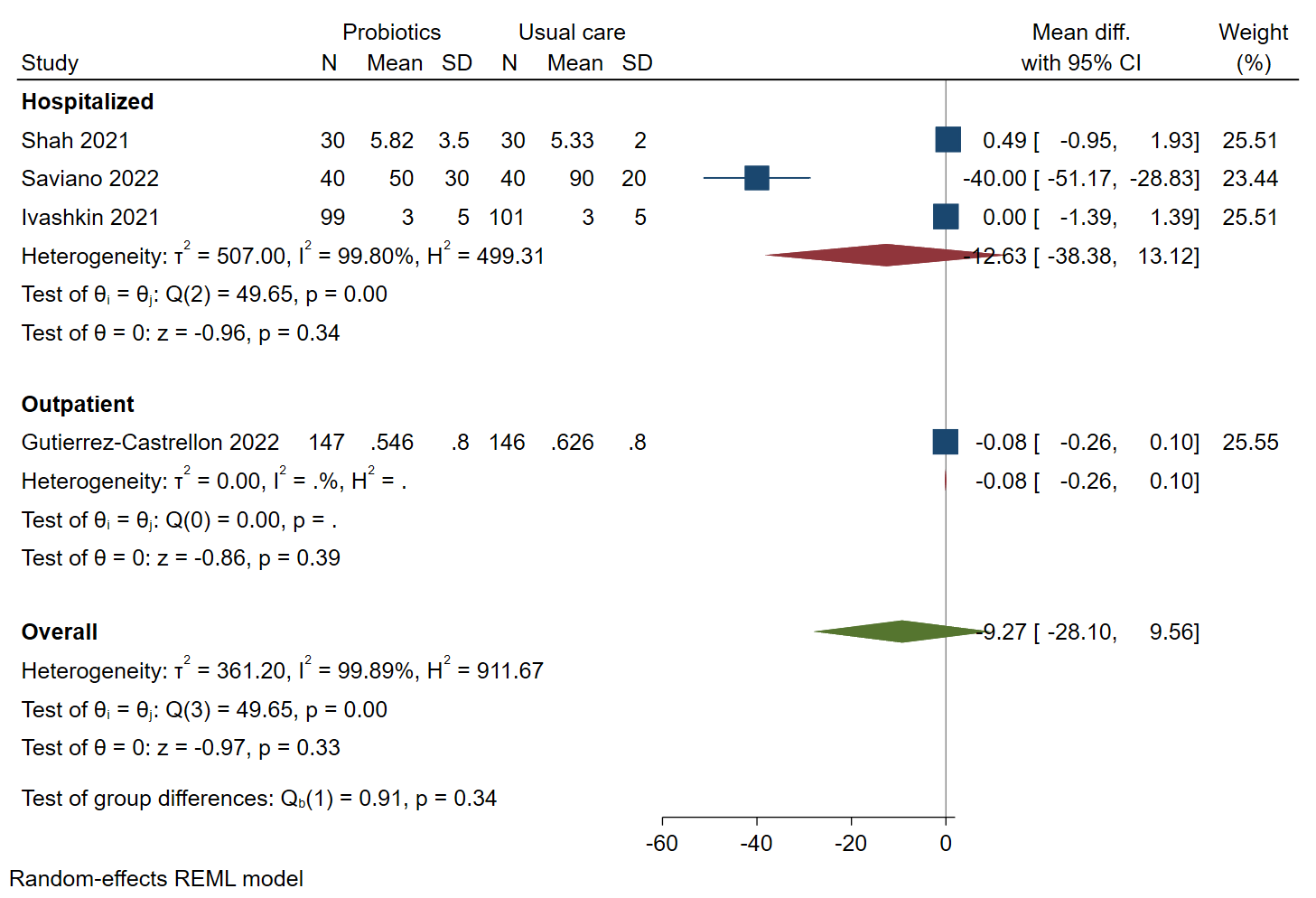

Supplement: S3 Fig — (TIFF) [file pone.0278356.s004.tiff]

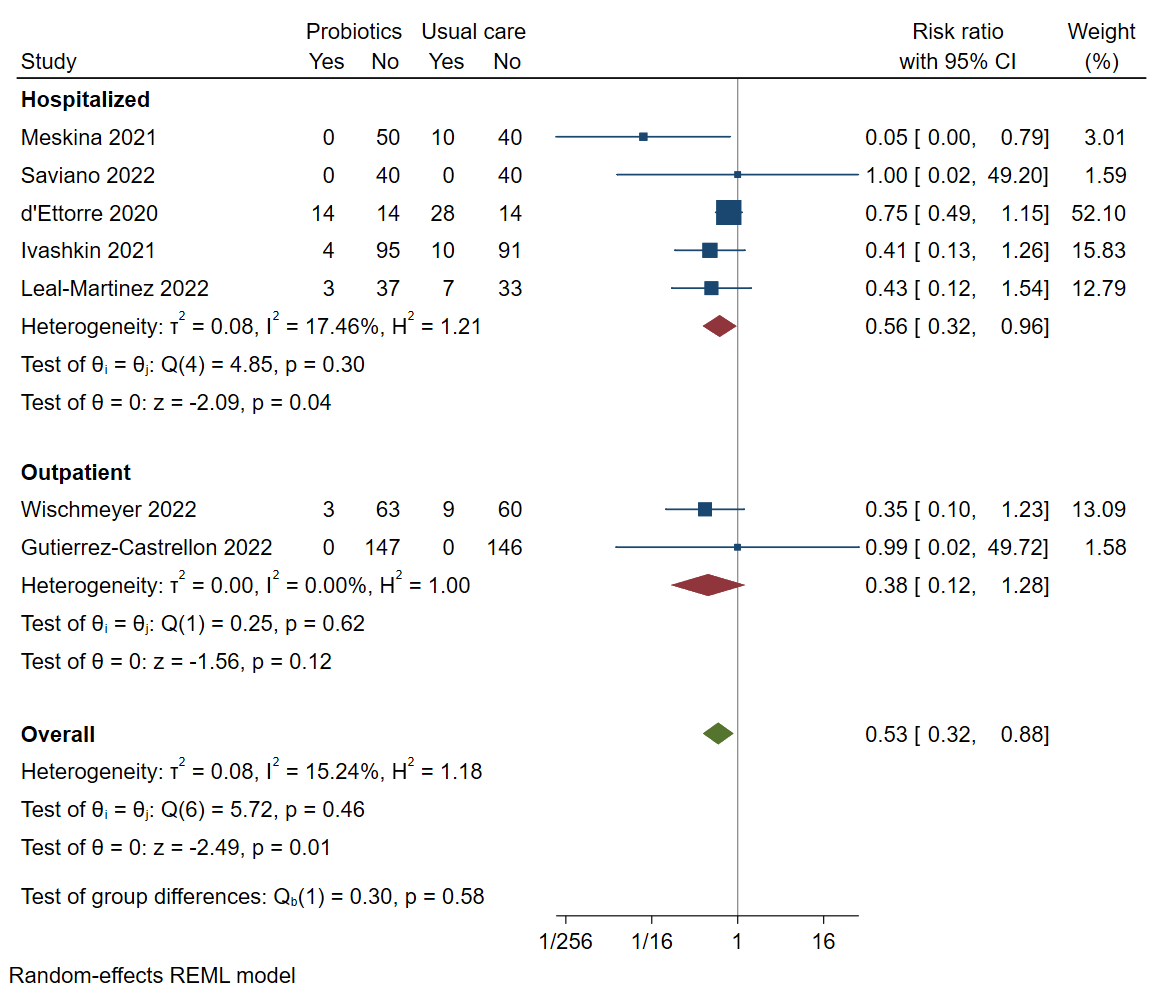

Supplement: S4 Fig — (TIFF) [file pone.0278356.s005.tiff]

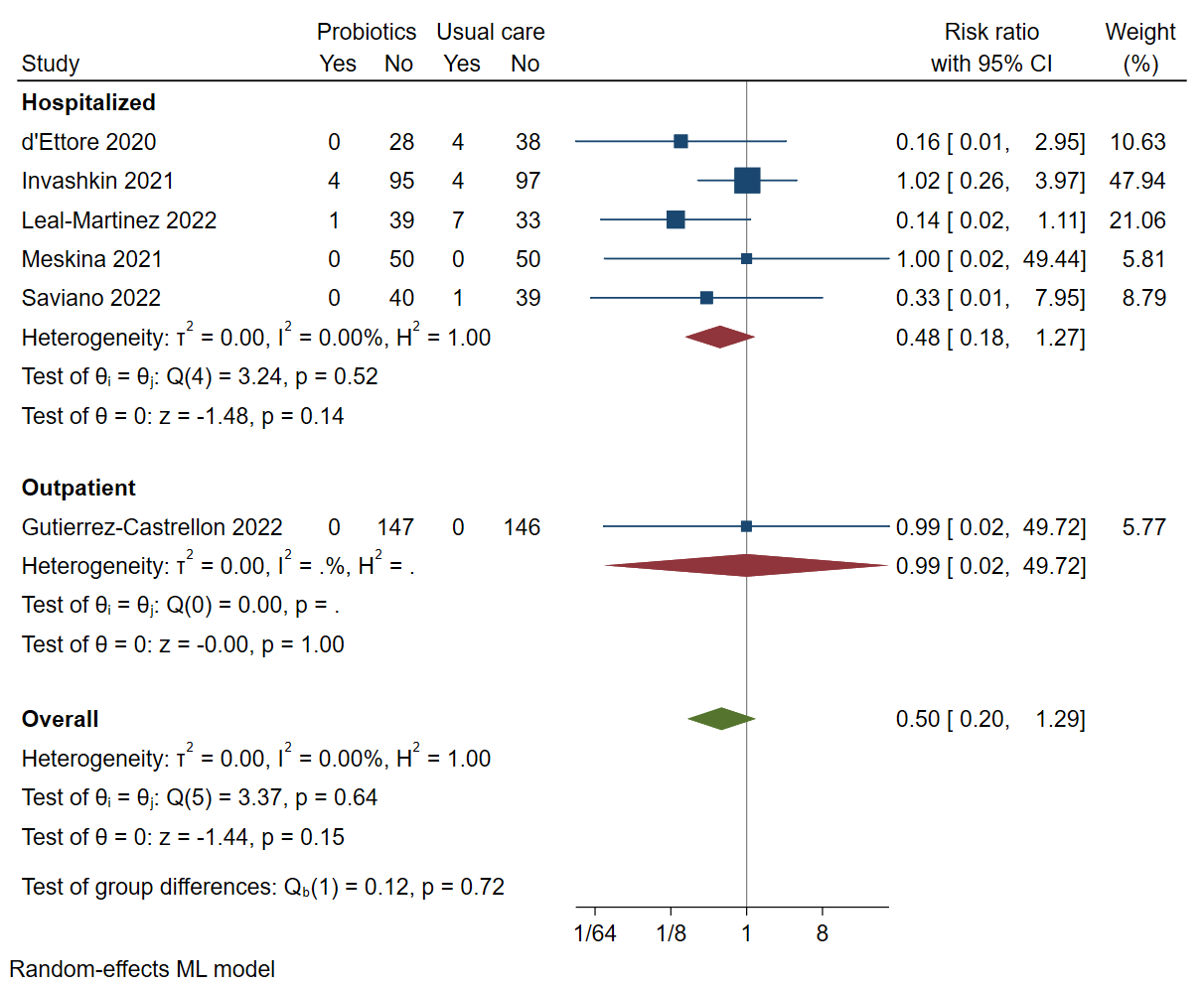

Supplement: S5 Fig — (TIFF) [file pone.0278356.s006.tiff]

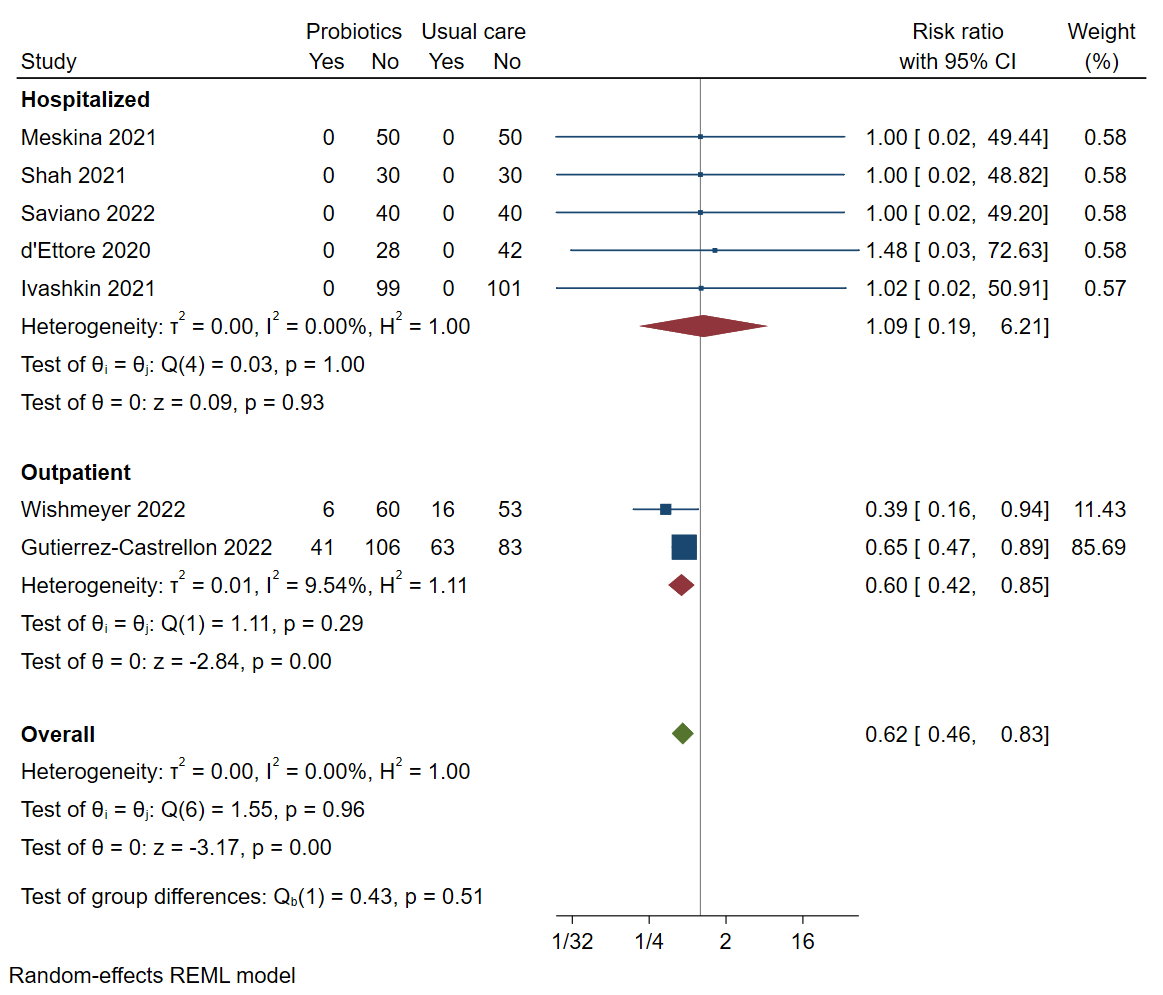

Supplement: S6 Fig — (TIFF) [file pone.0278356.s007.tiff]

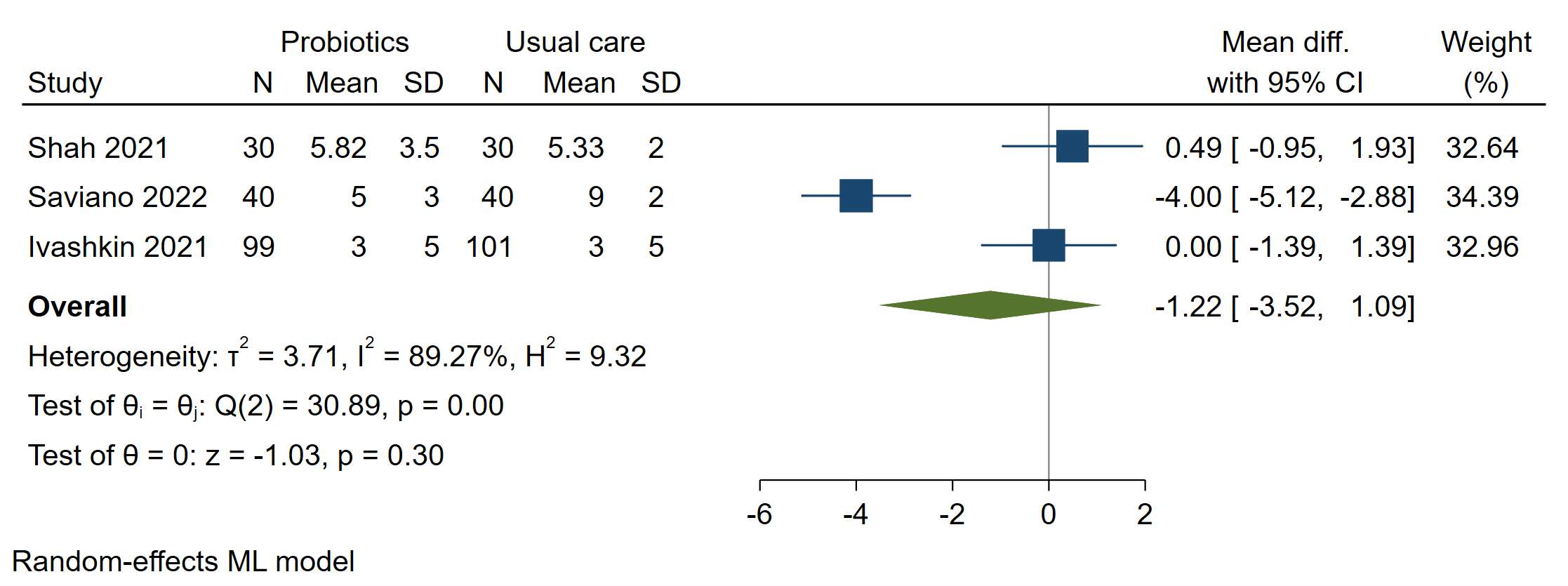

Supplement: S7 Fig — (TIFF) [file pone.0278356.s008.tiff]

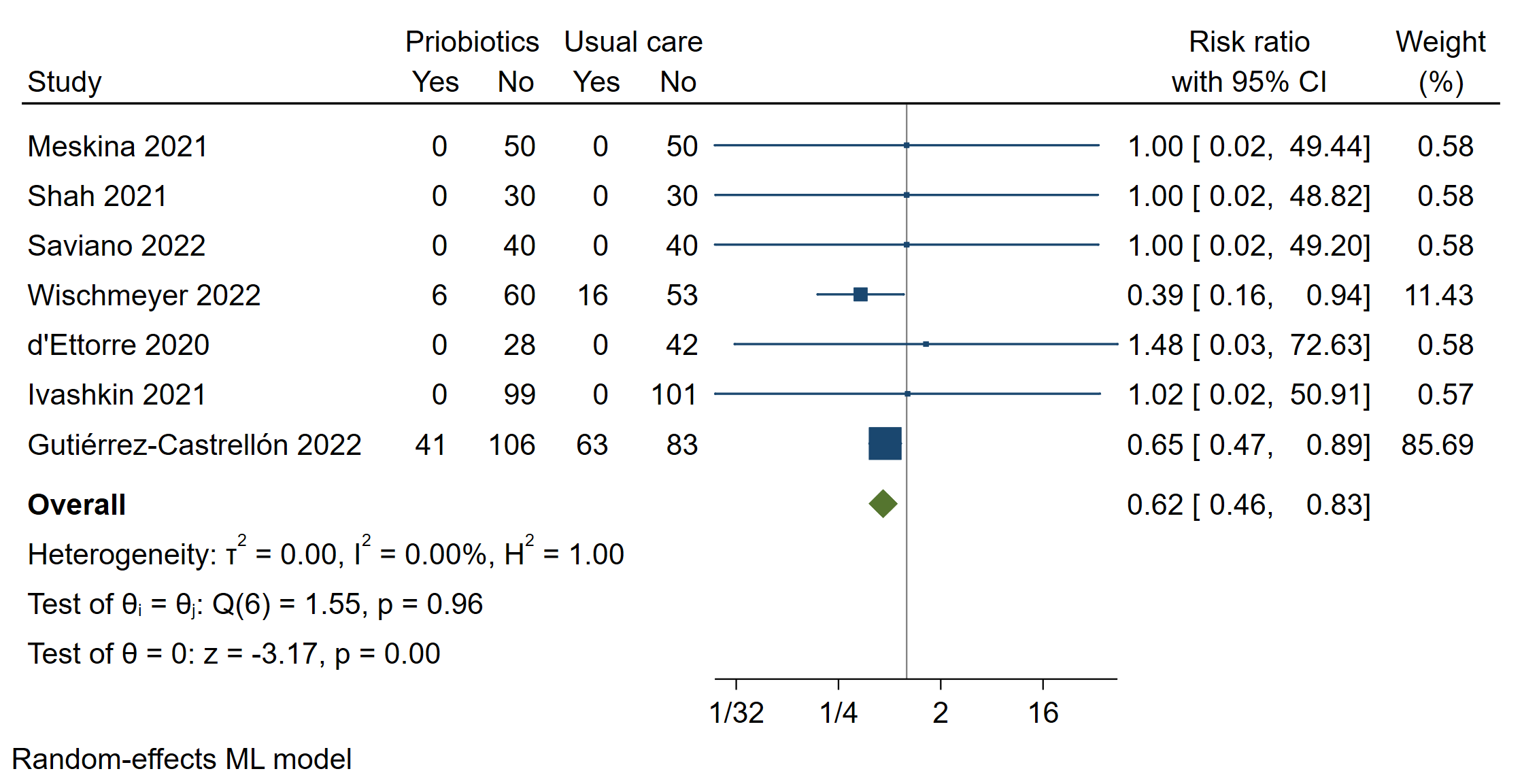

Supplement: S8 Fig — (TIFF) [file pone.0278356.s009.tiff]

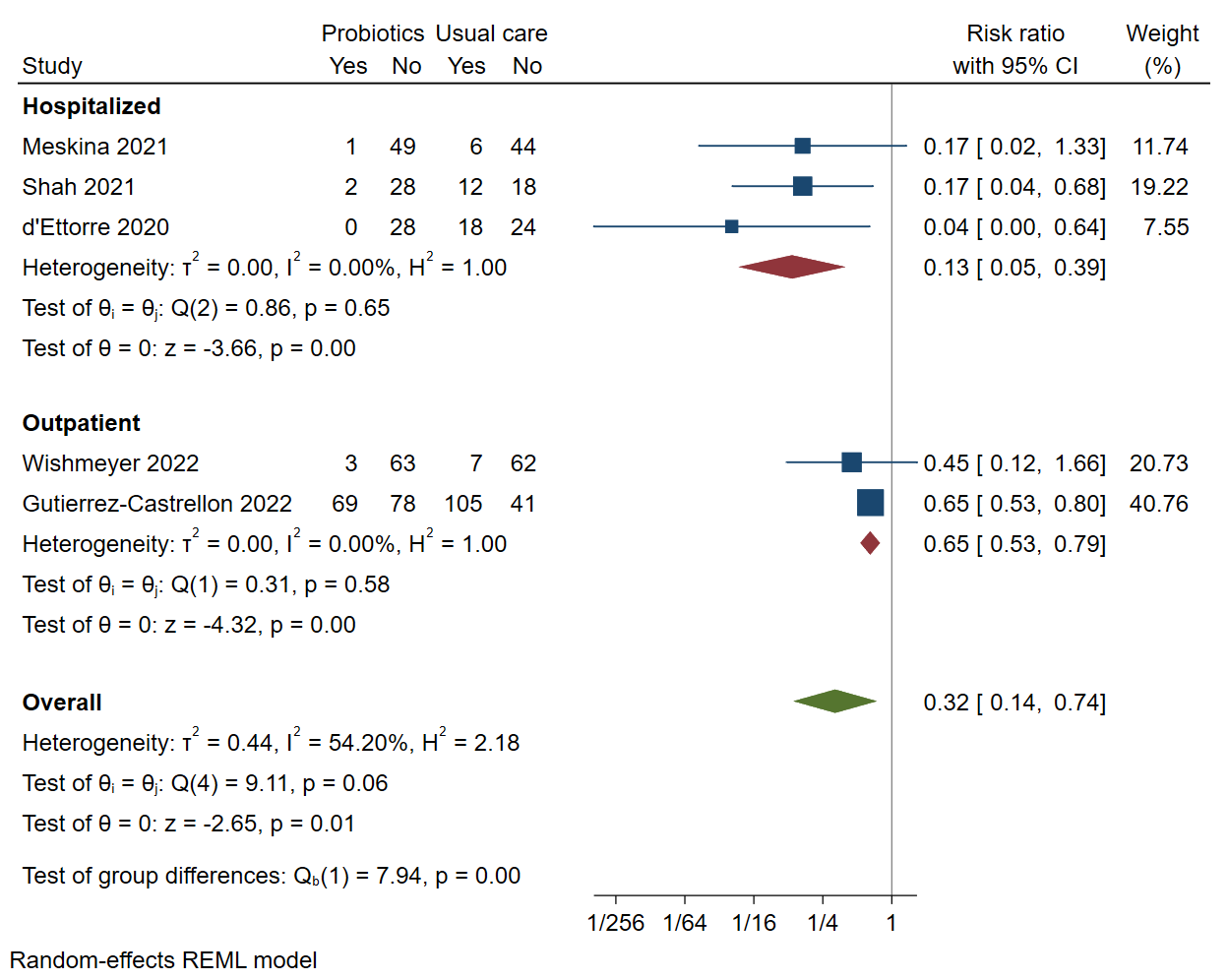

Supplement: S9 Fig — (TIFF) [file pone.0278356.s010.tiff]
